# Study on the Physical, Thermal and Mechanical Properties of SEBS/PP (Styrene-Ethylene-Butylene-Styrene/Polypropylene) Blend as a Medical Fluid Bag

**DOI:** 10.3390/polym14163267

**Published:** 2022-08-11

**Authors:** Satisvar Sundera Murthe, Srimala Sreekantan, Rabiatul Basria S. M. N. Mydin

**Affiliations:** 1 School of Materials and Mineral Resources Engineering, Universiti Sains Malaysia, Engineering Campus, Nibong Tebal 14300, Penang, Malaysia; 2 Oncological and Radiological Sciences Cluster, Advanced Medical and Dental Institute, Universiti Sains Malaysia, Bertam Kepala Batas 13200, Penang, Malaysia

**Keywords:** PVC-free, DEHP-free, SEBS, PP, blood bag, biomaterials

## Abstract

The presence of DEHP in PVC-based medical bags poses a significant health risk to patients undergoing blood transfusion. In order to fabricate safer medical fluid bag materials, the use of SEBS/PP polymer blend as a potential material was investigated. Polymeric blends with varying weight percentages of styrene-ethylene-butylene-styrene/polypropylene (SEBS/PP) were fabricated by melt mixing using an internal Haake mixer. The physical properties of the SEBS/PP polymer blends were investigated using differential scanning calorimetry (DSC), X-ray diffraction (XRD), and inductively coupled plasma–mass spectrometry (ICP-MS). In addition, measurements of the mechanical strength (tensile strength and Young’s modulus) as per ASTM 638, polymer hardness was tested using a durometer and swelling was analysed through water absorption and compared with commercial PVC-based blood bags. The results indicate that the SEBS/PP 50/50 blend has approximately similar characteristics as PVC-based blood bags. The SEBS/PP polymer blend possesses approximate tensile strength and Young’s modulus with values of 23.28 MPa and 14.42 MPa, respectively, to that of the conventional PVC blood bags. The results show that the SEBS/PP polymer blends have negligible zinc and aluminium migration with values of 1.6 and 2.1 mg/kg, respectively, and do not elute any harmful leachates, while the thermal studies indicate that the studied SEBS/PP materials are capable of withstanding steam sterilisation at 120 °C and cold storage below −40 °C. The investigated material can be utilized for medical fluid bags and contributes towards sustainable development goals, such as SDG 3 to ensure healthy lives and promote well-being, as well as SDG 12 to ensure sustainable consumption and production patterns.

## 1. Introduction

Red blood cells (RBCs) are an indispensable substance of life for human sustenance. The importance of RBCs is self-evident from the millions of RBC transfusions conducted annually to safely and speedily increase the supply of oxygen to the tissues to replace blood lost during haemorrhage or to increase a low haemoglobin concentration [1,2,3]. These transfusions are able to be carried out due to the disposable blood bags. The blood bags take on the roles of collecting, storing, transporting, and transfusing human blood and its various components [4]. The large majority of blood bags today are manufactured using polyvinyl chloride (PVC), a relatively rigid and brittle polymer valued for its inertness, durability, and resistance to heat, chemicals, abrasion, and kinking [5,6]. Due to the brittleness and rigidness of PVC, phthalate plasticisers are added to PVC blood bags to soften and increase the flexibility of the blood bags [6]. Plasticisers achieve the increased flexibility by inserting themselves between the PVC chains, thereby increasing the distances between the molecules without altering the microcrystalline structure of the polymer, which in turn increases the mobility of the PVC molecules. This leads to an increase in volume due to Brownian movement and renders the polymers more flexible by several degrees [6,7,8]. The most commonly utilised phthalate plasticiser for the vast majority of the commercial blood bags available on the market is di-2-ethylhexyl phthalate (DEHP).

However, it was discovered that DEHP leaches out from the walls of the PVC blood bag due to the lipophilic nature of DEHP and migrates preferentially to lipid-containing environments owing to the higher solubility of DEHP under such conditions [9]. A slow but steady constant release of DEHP is to be expected from plasticised PVC blood bags that come into contact with human blood that contains lipids [10]. The leached DEHP are then taken up by the RBCs [11]. Studies have shown that DEHP confers a preservative effect on blood by providing a protective effect on the RBCs’ membrane and cytosol [12,13]. DEHP also has been demonstrated to enhance the morphology, deformability, osmotic fragility, and microvesicle release of the stored RBCs. It was also discovered that RBCs infused with leached DEHP exhibit improvements in blood viscosity and show a lower haemolysis than RBCs that are bereft of DEHP contact [13]. However, these benefits of DEHP are mired by numerous negative health effects. DEHP may promote the release of pro-inflammatory cytokines [14]. Additionally, studies have shown that long-term exposure to monoethlyhexyl phathalate (MEHP), the primary metabolite of DEHP, has carcinogenic potential. DEHP is also suspected to partake in the disruption or modulation of the endocrine signalling involved in the regulation of neurodevelopment and reproduction [15,16]. Numerous studies have also shown that the harmful effects of DEHP can affect numerous organs including the liver, reproductive tract, kidneys, lungs, and heart [17]. Neonates are particularly at a higher risk of bearing the full brunt of DEHP hazards due to their small stature and higher exposure [18]. Moreover, the disposal of PVC bags leads to the emission of dioxin and greenhouse gases when incinerated and releases metal and toxic leachates when landfilled [19,20]. In response to these health hazards, numerous alternative plasticisers were studied to eradicate the disadvantages of DEHP [21,22,23]. The current works identify plasticisers such as DINCH, DEHA, DEHT, ATBC, TETM, and TOTM as the major alternative plasticisers for PVC blood bags. However, these plasticisers have flaws such as foul smell, high costs, and the inability to be steam-sterilised properly as well as not possessing adequate data on its long-term health effects [12,13,24].

These factors create the need for alternative PVC- and DEHP-free materials for the fabrication of medical fluid bags. Among the alternate materials are blends of thermoplastic polyurethane/polypropylene, thermoplastic polyurethane/polypropylene/ethylene-vinyl acetate, and polyolefins [4,5,25]. These PVC-free medical fluid bags also possess flaws such as relatively high Young’s modulus, inability to be adequately sterilised, poor mechanical strength, and the inability to be welded or sealed using high frequency RF (radio-frequency) welding [4,5,13]. These aforementioned studies also lack clear elucidation on the thermal properties of the medical fluid bags which is necessary for proper RBC storage. In this work, we have fabricated a polymer blend composed of SEBS and PP that seeks to overcome the limitations of the previous studies. This study outlines the results of the physical analysis of SEBS/PP blends as replacement for PVC blood bags. The SEBS and PP polymer duo has the capacity to be steam-sterilised and possesses low Young’s modulus, good mechanical strength, and thermal properties. In this study, SEBS/PP blends of different ratios were prepared; then, the physical, mechanical, thermal properties and ion migration were examined to compare them with those of commercially available blood bags. This work attempts to study the properties of SEBS/PP as alternative materials in order to remove the risks caused by DEHP in PVC-based blood bags.

## 2. Materials and Methods

### 2.1. Materials

SEBS (G1645V) was procured from Kraton Polymer (Wesseling, Germany) with a Shore A hardness of 35. It contains a polystyrene content of 11.5% to 13.5% and has an enhanced midblock (ethylene-butylene) for increased compatibility with polypropylene. Polypropylene random-heterophasic copolymer (Bormed SC820CF-11) was obtained from Borealis AG (Vienna, Austria) possessing a tensile strength of 50 MPa and melt flow rate of 3.9 g/10 min. Blood bag samples made from DEHP plasticised PVC were purchased from the Terumo Penpol Limited (Kerala, India). The samples were cut and washed once with 70% ethanol. The samples were then washed again with distilled water twice to remove any adhered impurities.

### 2.2. Preparation of Blends

The samples were weighed and measured prior to blending. The blending of components was performed in an internal mixer (Haake PolyDrive, Thermo Scientific, Karlsruhe, Germany) at 190 °C with a rotor speed of 50 rpm for 5 min. The SEBS/PP polymers were blended in compositions of 70/30, 50/50, and 40/60 wt%. The polymer blends in this experiment were successfully blended without the use of any polymer processing additives. A hydraulic hot press compression moulding machine (Model GT7014A, Gotech Testing Machines, Taichung City, Taiwan) was then utilised to cast the blended SEBS/PP polymers into monolayer films. A cast iron mould with a thickness of 0.5 mm acted as the mould for the polymer blend. After heating the blended material for 10 min to re-melt the polymer blend, it was compressed for 3 min at 190 °C. The mould containing the plastic sheet was then compressed and cooled for 3 min using a cooling plate to allow the polymer blend to take the form of a thin monolayer film. The moulds employed were coated with Teflon sheets to provide a non-adhesive surface to facilitate film removal.

### 2.3. Physical Characterisation

The mechanical properties (tensile strength, Young’s modulus, and elongation-at-break) of the SEBS/PP blends and commercial PVC film were assessed according to ASTM 638 at a room temperature of 23 °C in a Universal Testing Machine (Model UMH-50, Shimadzu, Japan) at a cross-head speed of 50 mm/min and compared with those of the commercial PVC blood bags. Dumb-bell shaped specimens as specified in ASTM 638 were prepared using a die cutter. A minimum of five samples were tested in each case to get accurate measurements. Additionally, the swelling property of the SEBS/PP 50/50 blend was examined by measuring the water absorption content after measuring the dry weight of the sample and then immersing it in purified water that was present in large excess compared to the size of the polymer. The swollen films were taken out of the water after 24 h and the surfaces dabbed with filter paper prior to measuring the wet weight of the samples. The swelling equilibrium was established until no further weight increase was observed. The swelling capacity was to be calculated using the following formula:

Swelling (%) = [(wet weight − dry weight)/dry weight] × 100

Morphology studies were conducted using a Field Emission Scanning Electron Microscope (FESEM) (Hitachi-Regulus SU8220, Tokyo, Japan). The SEBS/PP polymeric blends and commercial PVC film were cryogenically fractured using a mallet after immersing the films in liquid nitrogen for 30s. The fractured cross-section surface was coated with platinum and scanned using SEM.

### 2.4. Optical Characterisation

The optical properties of the polymers were assessed in order to allow medical practitioners to check for contamination and haemolysis due to storage lesions more easily as compared to conventional PVC-DEHP blood bags [26]. Visual detection of haemolysis in a unit is usually possible by observing the colour of the supernatant plasma. It is good practise to observe the colour of the plasma just before issue to avoid any inadvertent serious transfusion of a haemolysed blood unit. UV–vis absorption spectra were recorded with a Spectrophotometer UV-Vis (Shimadzu, Kyoto, Japan) within a range of 540–560 nm according to ASTM D1746-03, the standard test method for testing the transparency of plastic sheeting. Empty compartment (air) was used as the baseline.

### 2.5. X-ray Diffraction (XRD) Characterisation

The crystalline structure and XRD spectra were measured by means of the X-ray diffraction (XRD) method. A high-resolution X-ray diffractometer (Malvern Panalytical, Malvern, Worcestershire, UK) (with Cu K-alpha radiation (λ = 0.154184 nm) and a Ni filter with a generator voltage of 40 kV and a current of 30 mA was used. The radiation was measured with a proportional detector. The samples were measured in θ–2θ geometry over a range of 5° to 80°. All measurements were carried out at room temperature with a step size of 0.01° and a counting time of 5 s per data point. The crystalline phases were identified using the X’Pert High Score Plus software (v3.0e, Malvern Panalytical, Malvern, UK).

### 2.6. Differential Scanning Calorimetry (DSC) Characterisation

The thermal behaviour of SEBS/PP (70/30, 50/50, and 40/60) were studied using differential scanning calorimetry (Perkin-Elmer DSC 7, Boston, MA, USA) in a nitrogen atmosphere (50 mL/min) with a heating rate of 10 °C/min. In the first heating and cooling scans, the samples were heated from 25 to 190 °C in order to eliminate any previous thermal history. Then, the samples were cooled with liquid nitrogen from 190 to −70 °C at a cooling rate of 10 °C/min, followed by reheating to 190 °C for the second heating run [27].

### 2.7. Hardness Characterisation

The material hardness was determined by using a Durometer (Shore A). The measurements were taken at five different points on a single specimen of plastic sheet.

### 2.8. Inductively Coupled Plasma-Mass Spectrometry (ICP-MS) Analysis

The ICP-MS analysis was carried out by My CO2, an independent analytical testing laboratory. The analysis was carried out as medical fluid bag materials that come in contact with blood directly may also cause metal ions and elements originating from the processing of medical fluid bags to be leached out and migrated into blood. The ICP-MS method was utilised to assess the heavy metal migration from SEBS/PP 50/50 and PVC-DEHP. SEBS/PP 50/50 was selected as the representative of the SEBS/PP blends as it has the most approximate mechanical characteristics to the compared with commercial PVC-DEHP blood bags. Briefly, the metal amount content was determined using an inductively coupled plasma (ICP) equipped with mass spectrometer (MS) (Analytik Jena, Jena, Germany). The metals that were determined were 9 metallic elements, namely barium, cobalt, copper, iron, lithium, manganese, nickel, zinc, and aluminium to assess whether it falls within the restriction levels defined by EU No. 10/2011. The specific migration analysis of zinc and aluminium was conducted as zinc- and aluminium-based additives are commonly used for the processing of polymers. Each analysis was replicated three times. Heavy metal migration from the two polymer blends were soaked for 10 days in 3% acetic acid, olive oil, and 10% ethanol, respectively, and analysed.

## 3. Results and Discussion

### 3.1. Mechanical Characterisation

Table 1 shows the mechanical properties of the polymer blends and the commercial PVC blood bags with the standard deviations provided. The materials’ tensile behaviour provides critical information about their tensile strength, which indicates the blended materials′ ability to safely contain the required amount of blood without rupturing or bursting. The tensile tests also provide data on Young’s modulus, revealing the stiffness of the material and, by extension, a view into the flexibility of the blended materials. This set of modulus data ascertains the capabilities of the blended material to withstand rough mechanical handling without tearing.

Table 1 clearly shows that the tensile strength and Young’s modulus of the blended polymers decrease with an increase in SEBS content, whereas the elongation-at-break of the polymer blends increase with an increase in SEBS content. This indicates that the inclusion of PP into SEBS leads to an increase in tensile strength and Young’s modulus. The variation can be primarily attributed to the antagonistic intrinsic nature of the polymers; SEBS is a thermoplastic elastomer with excellent flexibility and elasticity, whereas PP exhibits high strength and high Young’s modulus but possesses paltry impact resistance [28,29,30]. These characteristics culminate in the toughening of SEBS by PP and reduction of stiffness of PP by SEBS, thus providing the SEBS/PP polymer blend with the desired mechanical properties for a viable medical fluid bag. Similar results can be observed in other studies as well [31,32]. The weak mechanical properties of 100% SEBS can be attributed to the partial and selective hydrogenation process of styrene-butadiene-styrene (SBS), which is used to synthesise SEBS elastomers that are thermally stable at high temperatures [33]. These major mechanical weaknesses of SEBS necessitate for SEBS polymers to be blended with other polymers to enhance its mechanical attributes. The elastic modulus and tensile strength are increased with the addition of PP as the mechanical attributes of PP are relatively higher than those of SEBS. The blending of SEBS with PP is touted as a rational one as evidenced by a study which demonstrated that the interfacial adhesion between the SEBS and PP matrix contributes to good impact properties. The good interfacial adhesion between the two polymers is precipitated by the segmental diffusion of ethylene-butylene (EB) mid-blocks with PP matrix. Furthermore, owing to this segmental diffusion, the common weaknesses of polypropylene, such as poor impact resistance stemming from its high crystallinity and relatively high glass transition, are overcome by SEBS elastomers that promote relatively higher impact strength and elongation-at-break as well [33]. The SEBS/PP blend which comprises of elastomeric and semicrystalline polymers shows an increase in Young’s modulus as the decreased presence of elastomeric phases increases the stiffness of the polymer blend. While the rise in Young’s modulus in not prominent, the differences are relevant in order to create a medical fluid bag that is in close approximation to PVC-DEHP blood bags. It is important to note that the addition of PP to SEBS does not result in uniform increase in Young’s modulus.

Similar to the Young’s modulus, the tensile strength of the SEBS/PP polymer blends increased linearly with decreasing SEBS content. The SEBS/PP 40/60 wt% exhibited the highest tensile strength of 32.64 MPa, whereas SEBS/PP 70/30 wt% showed the lowest tensile strength 0.97 MPa. This large disparity can similarly be attributed to the decreasing percentage of elastomer which is replaced with PP that has a higher tensile strength. The commercial blood bags composed of PVC and DEHP showcased a tensile strength of 21.08 MPa, a Young’s modulus of 5.59 MPa, and an elongation-at-break of 765.17%. The impressive tensile strength, coupled with a low Young’s modulus, is only made possible through the use of DEHP and other processing oils such as epoxidized soybean oil [34]. As a thumb rule, the flexibility of plastic can be increased through the use of plasticisers, but at the cost of the strength of the material [35]. The SEBS/PP composition of 50/50 represents the optimal composition for a PVC-free medical fluid bag. It is the optimal choice considering that it has an approximate tensile strength and a Young’s modulus similar to the commercial blood bag with values of 23.28 MPa and 14.42 MPa, respectively. It is also important to note that although the values deviate slightly from the commercial blood bag, the tensile strength falls within the permitted range of 20–30 MPa while the Young’s modulus falls within the range of 5–15 MPa [36,37]. Interestingly, the majority of the 100 wt% SEBS exhibited thermoplastic elastomeric behaviour by exceeding the tensile test limit of 2400% elongation, indicating overwhelmingly superior elongation capacity as compared to 100 wt% PP, which has a respectable elongation-at-break of 1642.60%.

The addition of even a low amount of 40 wt% SEBS shows a marked drop of mechanical properties with a tensile strength of 24.93 MPa and a Young’s modulus of 32.64 MPa as compared to those of pure 100 wt% PP, which has a tensile strength of 32.55 and a Young’s modulus of 321.56 MPa. The drop in Young’s modulus is especially prominent. The significant drop can be attributed to the 100 wt% SEBS that has a meagre tensile strength of 7.06 MPa and an even staggeringly lower Young’s modulus of 0.43 MPa. This extent on reduction of mechanical properties can also be attributed to the blend morphology wherein blends having coarser dispersions exhibit more reduction in properties [4]. Commonly, increased interfacial tension leads to an increase in the tendency of coarsening due to coalescence [38]. Furthermore, a study has shown that the addition of even a minute percentage of SEBS into PP causes a marked increase in PP spherocrystals but shows a decrease in their sizes [30]. The addition of additional SEBS into the polymeric blend logically results in the increase in the number of spherocrystals and its inevitable reduction in size. These can be ascribed to SEBS which acts as the nucleating agent thereby forming more nucleating sites with PP. The study has shown that spherocrystals in 30 wt% of SEBS can barely be seen and provides significant flexibility to the polymeric blend. The decrease in the size of the spherocrystals is also accompanied by the lessening of their completeness. Additionally, another study has shown that SEBS elastomer particles are well dispersed with PP matrix in irregular forms with a narrow size distribution and showcased a two-phase system which in turn promoted the strain-rate-sensitivity of SEBS/PP blends [39]. Moreover, the affinity of the EB midblock of SEBS with PP matrix can also serve to decrease the modulus of SEBS/PP blends with the addition of SEBS due to the substitution of PP matrix by soft SEBS elastomer (replacement of the plastic component with the elastomeric one) [32,39]. The substitution also results in the elongation of the polymeric blends to increase.

Furthermore, the elongation-at break-increases with the addition of SEBS due to the increase in ductility of the polymeric blends [32]. This stems from an increase in interactivity between PP and SEBS, particularly the EB blocks of SEBS. Therefore, the ductility increases with the increase in elastomer composition and thereby increase the elongation-at-break of the polymeric blends. The increase in ductility of polymer blends in spite of the reduction of the tensile strength can be attributed to a higher deformation level. The increase in SEBS weight percentage increases the ductile behaviour of the polymer blends and plastically deforms them until breakage [40]. Commonly, an increase in elongation-at-break is presumed to be an indication of increased compatibility between polymer phases [41]. This is supported by FESEM analysis, which exhibits good compatibility between SEBS and PP. Table S1 provided in the supplementary data shows the mass of the SEBS/PP 50/50 specimens before and after immersion in distilled water. No polymer swelling was observed in the SEBS/PP 50/50 polymer. This indicates that the polymer blend has water barrier properties that prevent water vapour from entering or escaping the confines of the medical fluid bag structure.

**Figure 1:**
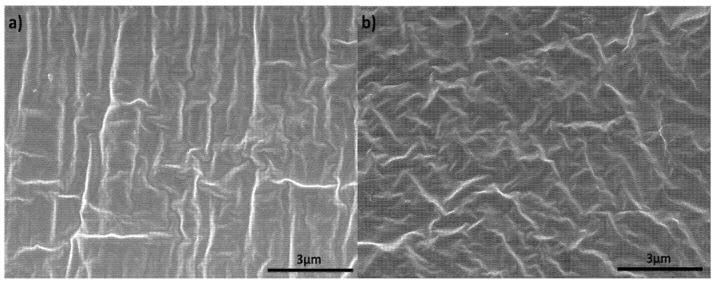
FESEM cross-section image of (**a**) SEBS/PP 50/50 polymer blend and (**b**) commercial PVC-DEHP film.

The morphology of the SEBS/PP 50/50 polymer blends and commercial PVC-DEHP films were observed using FESEM. Figure 1a below shows the cryo-fractured cross-section of SEBS/PP 50/50, whereas Figure 1b shows the cryo-fractured cross section of the commercial PVC film. The cross-section of both samples exhibits a rough structure originating from ductile material fracture [42]. In addition to that, Figure 1a shows thread-like fibres with continuous structure devoid of any dispersed domain [43,44]. This supports the aforementioned mechanical results that SEBS/PP polymer blends have good compatibility and miscibility owing to the EB block of SEBS which is miscible with the PP matrix. The high miscibility is also supported by the DSC results discussed later, which only show a singular glass transition temperature [45]. Highly miscible blends exhibit only a single glass transition peak at some intermediate value between the glass transition of the two-component polymer. Furthermore, Figure 1b shows a dense homogenous structure [46]. The rough surface of the commercial PVC-DEHP cross-section indicates a ductile nature. The presence of the DEHP plasticiser diminishes the brittle nature of pure PVC.

### 3.2. Optical Analysis

**Figure 2:**
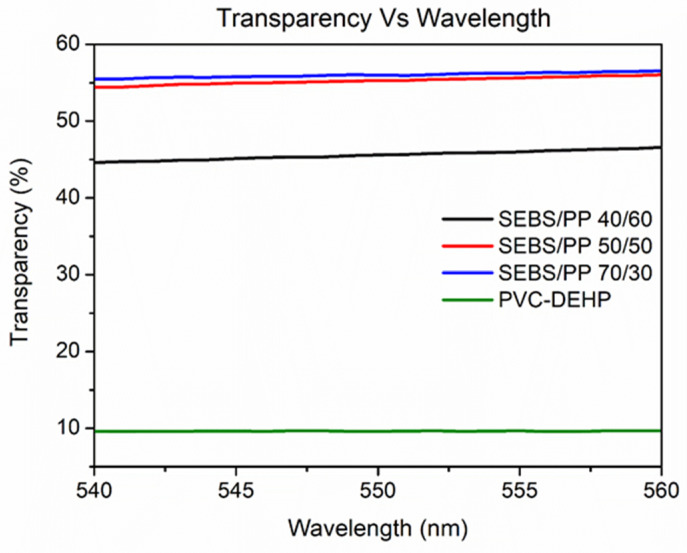
Transparency percentage vs wavelength plot of SEBS/PP polymer blends and PVC-DEHP polymer film.

The conventional PVC-DEHP material exhibits the lowest transparency percentage with a meagre value of 9.7%, whereas the transparency value of the optimal SEBS/PP 50/50 medical fluid bag film is 56%. While an increase in SEBS content from 40 to 50 wt% leads to an increase of 10 percent in transparency, an increase from 50 to 70 wt% only leads to an increase of 0.5% in transparency. This indicates that the SEBS/PP polymer blend reaches saturation levels of transparency after 50 wt% of SEBS. Table 2 clearly outlines the transparency percentages of the SEBS/PP blends and compares them to the commercial PVC blood bag. In spite of the conventional PVC blood bag material having approximate mechanical characteristics to SEBS/PP 50/50, the transparency of SEBS/PP 50/50 and the benefits it confers to the healthcare personnel are better. Additionally, Figure 2 shows the transparency percentage of the SEBS/PP polymer blends to showcase the difference in optical transparency between the polymers. The addition of SEBS content to the polymeric blend results in a higher transparency, thus increasing the visibility of the material.

### 3.3. X-ray Diffraction (XRD) Analysis

**Figure 3:**
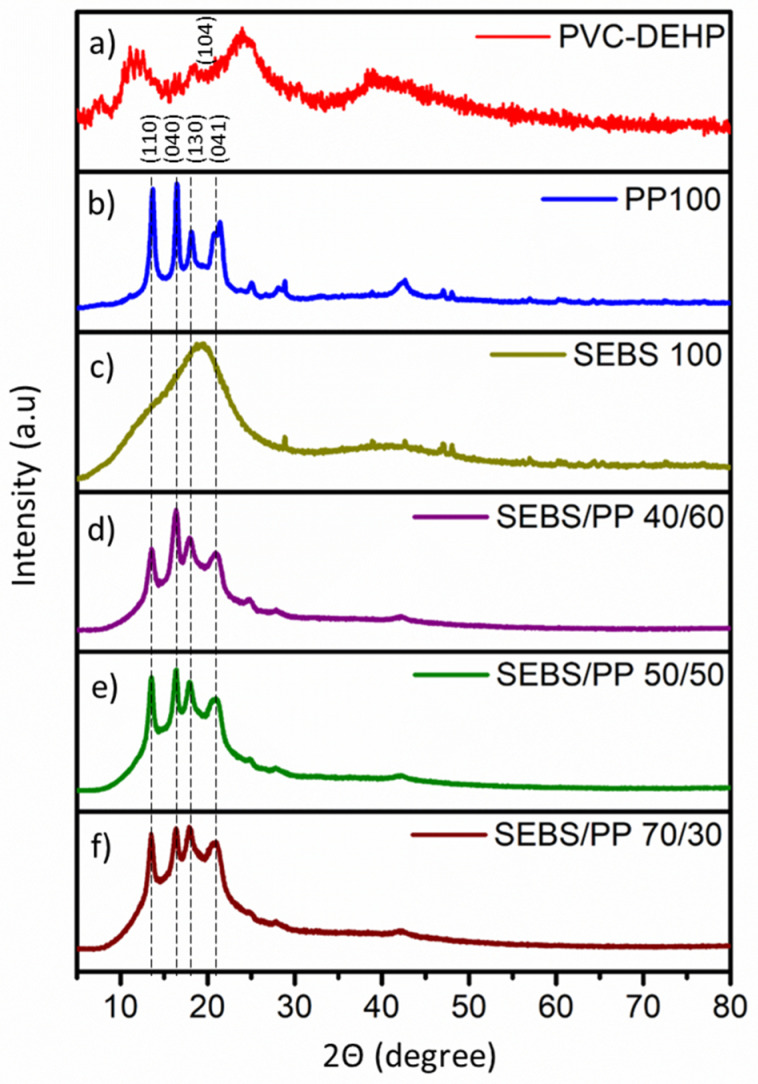
XRD spectra of (**a**) commercial PVC-DEHP film, (**b**) 100 wt% PP, (**c**) 100 wt% SEBS, (**d**) SEBS/PP 40/60, (**e**) SEBS/PP 50/50, and (**f**) SEBS/PP 70/30 polymer blend.

The crystallinity and plane orientation of the synthesized SEBS/PP polymer blend at different weight percentages were analysed via XRD. Figure 3a shows the XRD spectra for PVC-DEHP, whereas Figure 3b,c shows the XRD spectra of 100 wt% PP and 100 wt% SEBS and SEBS/PP polymer blends. Additionally, Figure 3d–f shows the XRD spectra for SEBS/PP polymer blends. SEBS being an amorphous material, it shows a broad curve and does not exhibit any sharp peaks. The 100 wt% SEBS forms a broad peak at 18.3° (130) with some minor peaks. The isotactic PP exists in three different crystal forms, namely α, β, and γ which entirely depend on the crystallization condition or additions of specific fillers [47]. The major peaks seen in the SEBS/PP polymer blends spectra correspond to the peaks of PP, with diffraction peaks at 14.1° (110), 16.9° (040), 17.5° (130), and 21.8° (041) to α form PP crystals [47]. These diffraction peaks indicate that the α form crystals dominate in the SEBS/PP polymers blends.

The intensity of the peaks at (110) and (040) plane decreases as the SEBS weight percentage within the polymer blend increases, indicating a decrease in the degree of crystallinity of the samples [48,49]. The polymer blend peaks are decreased slightly in intensity due to the presence of broad peaks arising from the addition of large amounts of amorphous SEBS. On the other hand, the PVC-DEHP crystalline peaks can be observed at 16.3°, 17.8°, 24.8°, and 39°, observed in previous studies in line with the amorphous nature of PVC [50,51,52,53]. The XRD spectra of PVC-DEHP also supports the presence of impurities in its structure. Additionally, the PVC-DEHP is abundant with the presence of impurities arising from additive substrates utilised during fabrication as indicated by the peaks at 11.1°, 18.6°, and 28.3° [54]. The peak at 23.9° can be attributed to the presence of CaCO_3_, a filler additive added into the PVC matrix [54]. The blends studied in this work solely consist of SEBS and PP. The absence of other phases in the SEBS/PP blends demonstrates that our material is safe.

### 3.4. Differential Scanning Calorimetry (DSC) Analysis

Table 3 lists the observed thermal transition of the materials. The results indicate that the crystallisation temperature of SEBS/PP 50/50 is achieved at a temperature of 87.09 °C. The most important observation to take from this study is that the melting temperature of the SEBS/PP 50/50, which stands at 144.70 °C, exceeds the required temperature for conventional blood bags. Additionally, all variations of SEBS/PP polymer blends in this study exceed the sterilisation temperature of 120 °C. Furthermore, to prove that this material is also capable of withstanding steam sterilisation, the SEBS/PP polymer blends were subjected to an autoclaving process where the plastic blend was in direct contact with super-heated water [55]. Figure S1a shows the SEBS/PP 50/50 specimen before steam sterilisation while Figure S1b shows the specimen after steam sterilisation. The results reveal that there were no discernible alternations to the polymer blend, be it colouration, transparency, shrinkage, or hardness. The hardness of the specimen before and after sterilisation was measured using a durometer (Shore A), whereas the transparency was measured using a UV-Vis spectrophotometer as per ASTM 1746-03.

Additionally, the SEBS/PP polymer blends are also capable of withstanding cold storage at sub-freezing temperatures, as evidenced by the glass transition temperature with a value of −40.72 °C. The rest of the SEBS/PP polymer blends possess glass transitions temperatures lower than −40 °C as well. Although the common storage temperature of blood bags is just between the range of 0 to 6 °C, rarer blood types are often frozen to allow prolonged storage so that they may be used when the occasion arises [56]. It must be noted that PVC-DEHP blood bags can be used at low temperatures due to the use of plasticisers and processing oils, which are harmful to both human health and the environment.

### 3.5. Hardness Analysis

Table 4 shows the measured material hardness (Shore A) values. The measurements clearly reveal that the hardness of the polymer blend decreases with an increase in SEBS content. These results support the tensile strength and Young’s modulus measurements discussed earlier. The low hardness of SEBS is the major factor which contributes to the reduction in polymer hardness [30,57]. The reduction in hardness can also be attributed to the compatibility of SEBS with PP. The middle block regions of the SEBS chains are capable of fusing easily to the PP matrix [58]. In addition, 100 wt% PP are relatively hard polymers and this necessitates the use of large amounts of synthetic rubber-based polymers such as SEBS to fabricate new polymer blends [59]. The hardness of SEBS/PP 50/50 with a value of 88.9 is approximately similar to the hardness of PVC-DEHP which has a value of 82.9. This difference of 6 Shore A hardness is negligible, and this factor affirms the proposition that the SEBS/PP 50/50 is suitable to be used as medical fluid bags. Additionally, the hardness of SEBS/PP 50/50 satisfies the requirement for tubing to be welded to the SEBS/PP material [58]. This strongly supports the proposition that the SEBS/PP polymer blend is capable of being welded together through radio-frequency welding.

### 3.6. Inductively Coupled Plasma-Mass Spectrometry (ICP-MS) Analysis

The ICP-MS characterisation highlighted the presence of metals. In the analysis, metals are expected to be found at permissible levels as it is intended for use as medical fluid bag materials. In the SEBS/PP and PVC-DEHP used in this work, a number of metals were found with differing concentrations. Table 5 shows the migration of metal from SEBS/PP 50/50 and PVC-DEHP. In the medical bag sector, different types of additives are commonly added into the polymers to obtain specific mechanical performance or optical behaviours [60]. In particular, stabilisers are the major sources of metals, such as zinc stearate and zinc oxide [61,62]. Additionally, various lubricant additives are added during fabrication to ease processing. The detected leaching amounts of 9 metallic elements (barium, cobalt, copper, iron, lithium, manganese, nickel, zinc, and aluminium) of both polymer blends were lower than the restriction levels defined by EU No. 10/2011, but the blends showed marked variation for specific metals [63]. The SEBS/PP 50/50 polymer blend film shows that the most leached element was zinc, followed by aluminium with concentrations of 1.6 and 2.1 mg/kg, respectively. The specific migration of the other metals was not detectable, owing as well to their non-existence or minute presence. The overall migration of metals from the SEBS/PP polymer blend was not detectable as well and was lower than the permitted limit of 10 mg/dm^2^. Similarly, the PVC-DEHP film also demonstrated that the most leached element was zinc, followed by aluminium with concentrations of 6 and 2.8 mg/kg, respectively. The relatively higher amount of zinc and aluminium in PVC-DEHP as compared to SEBS/PP 50/50 can be attributed to the additives incorporated in PVC-based blood bags during processing [64]. The presence of metallic impurities from antioxidants, mixed metal salt blends from heat stabilizers, and slip agents in the form of metallic stearates such as zinc stearate may have contributed to the relatively larger presence of metals in PVC-DEHP [61,62]. In comparison, SEBS/PP 50/50 has a relatively lower metal migration as no additives were used in the fabrication of the polymer blend. This SEBS/PP 50/50 polymer blend is relatively safer for use as a medical fluid bag as compared to PVC-DEHP. The XRD results which indicate the absence of impurities in the SEBS/PP polymer blends also support the ICP results that show no marked metal migration.

## 4. Conclusions

The results of this work clearly show that the SEBS/PP 50/50 polymer blends have the potential to be utilised as medical fluid bags, as the SEBS/PP composition has similar characteristics to PVC-DEHP-based blood bags. The tensile strength and Young’s modulus of the SEBS/PP 50/50 polymer blend is the only composition which has approximate values to that of PVC-DEHP blood bags. The XRD result shows that the SEBS/PP polymer blends do not have impurities in their composition. Moreover, the SEBS/PP polymer blends do not elute any harmful leachates due to the lack of impurities, as evidenced by the ICP-MS results. The elution of metals from the SEBS/PP was relatively lower than that of PVC-DEHP, thus confirming its safety. The thermal studies indicate that the studied SEBS/PP materials are capable of withstanding steam sterilisation at 120 °C and cold storage below −40 °C. The hardness assessment of the material validates the flexibility of the material and showcases that the material can be welded together for sealing purposes through radio-frequency welding. The SEBS/PP polymer blend properties suggest that it can be fabricated into PVC-free medical fluid bags and serve as a potential alternative to current commercial blood bags.

## Figures and Tables

**Table 1:** Tensile strength, Young’s modulus, and elongation-at-break of SEBS/PP polymer blends and PVC-DEHP.

Polymer Material	Polymer Composition (wt%)	Tensile Strength (MPa)	Young’s Modulus (MPa)	Elongation-at-Break (%)
SEBS (one specimen only)	100	7.06	0.43	2382.49
SEBS/PP	70/30	15.52 ± 1	0.97 ± 0.1	637.9 ± 5.3
SEBS/PP	50/50	23.28 ± 1.1	14.42 9 ± 0.9	121.2 ± 6.6
SEBS/PP	40/60	24.93 ± 1.2	32.64 ± 1.8	88.91 ± 7.3
PP	100	32.55 ± 6.2	321.56 ± 4.6	1886.7 ± 8.9
PVC-DEHP	N/A	21.08 ± 0.8	5.59 ± 0.5	874.48 ± 9.7

**Table 2:** Transparency of SEBS/PP polymer blends.

Polymer Material	Polymer Composition (wt%)	Transparency (%)
SEBS/PP	40/60	46.6
SEBS/PP	50/50	56
SEBS/PP	70/30	56.5
PVC-DEHP	N/A	9.7

**Table 3:** DSC data of SEBS/PP polymer blends.

Polymer Material	Polymer Composition (wt%)	T_c_ (°C)	T_m_ (°C)	T_g_ (°C)
SEBS/PP	40/60	94.50	142.15	−43.76
SEBS/PP	50/50	87.09	144.70	−40.72
SEBS/PP	70/30	80.21	142.84	−43.04

**Table 4:** Hardness (Shore A) of SEBS/PP polymer blends.

Polymer Material	Polymer Composition (wt%)	Hardness (Shore A)
SEBS/PP	40/60	91.9
SEBS/PP	50/50	88.9
SEBS/PP	70/30	76.5
PVC-DEHP	N/A	82.9

**Table 5:** Migration of specific metals from SEBS/PP 50/50 and PVC-DEHP polymer blend.

Specific Metal Migration	Polymer Composition	
	SEBS/PP 50/50 (mg/kg)	PVC-DEHP (mg/kg)
zinc	1.6	6.0
aluminium	2.1	2.8
barium	ND < 0.3	ND < 0.3
cobalt	ND < 0.05	ND < 0.05
copper	ND < 0.3	ND < 0.3
iron	ND < 0.2	ND < 0.2
lithium	ND < 0.5	ND < 0.5
manganese	ND < 0.3	ND < 0.3
nickel	ND < 0.03	ND < 0.03

## Data Availability

The data presented in this study are available on request from the corresponding author.

## Supplementary Materials

The following supporting information can be downloaded at: https://www.mdpi.com/article/10.3390/polym14163267/s1, Figure S1: Figure of the SEBS/PP 50/50 specimen (a) before steam sterilisation and (b) after steam sterilisation.; Table S1: Mass of the SEBS/PP 50/50 specimens before and after immersion in distilled water.

## References

[1] World Health Organization, International Federation of Red Cross, Red Crescent Societies (2010). “Towards 100 % Voluntary Blood Donation a Global Framework for Action”, World Health Organization. https://apps.who.int/iris/handle/10665/44359.

[2] National Clinical Guideline Centre (UK) (2015). “Blood Transfusion: NICE Guideline NG24”, National Institute for Health and Care Excellence (NICE), Nov. https://www.nice.org.uk/guidance/ng24/evidence/full-guideline-pdf-2177160733.

[3] Liumbruno G.M., Bennardello F., Lattanzio A., Piccoli P.L., Rossetti G. (2008). Recommendations for the transfusion of red blood cells. Blood Transfus..

[4] Ajili S.H., Ebrahimi N.G., Khorasani M.T. (2003). Study on thermoplastic polyurethane/polypropylene (TPU/PP) blend as a blood bag material. J. Appl. Polym. Sci..

[5] Ajili S.H., Golshan E.N., Khorasani M.T. (2003). Studies on TPU/PP Blend and Comparing it with PVC Used as Blood Bag. Iran. Polym. J..

[6] Simmchen J., Ventura R., Segura J. (2012). Progress in the Removal of Di-[2-Ethylhexyl]-Phthalate as Plasticizer in Blood Bags. Transfus. Med. Rev..

[7] Romero-Bastida C.A., Martín-Polo M.O., Velazquez G., Torres J.A. (2004). Torres, Effect of Plasticizer, pH and Hydration on the Mechanical and Barrier Properties of Zein and Ethylcellulose Films. Cienc. Tecnol. Aliment..

[8] Rahman M., Brazel C.S. (2004). The plasticizer market: An assessment of traditional plasticizers and research trends to meet new challenges. Prog. Polym. Sci..

[9] Erythropel H.C., Maric M., Nicell J.A., Leask R.L., Yargeau V. (2014). Yargeau, Leaching of the plasticizer di(2-ethylhexyl)phthalate (DEHP) from plastic containers and the question of human exposure. Appl. Microbiol. Biotechnol..

[10] Greiner T., Volkmann A., Hildenbrand S., Wodarz R., Perle N., Ziemer G., Rieger M.A., Wendel H., Walker T. (2011). DEHP and its active metabolites: Leaching from different tubing types, impact on proinflammatory cytokines and adhesion molecule expression. Is there a subsumable context?. Perfusion.

[11] Melzak K.A., Uhlig S., Kirschhöfer F., Brenner-Weiss G., Bieback K. (2018). The Blood Bag Plasticizer Di-2-Ethylhexylphthalate Causes Red Blood Cells to Form Stomatocytes, Possibly by Inducing Lipid Flip-Flop. Transfus. Med. Hemother..

[12] Bicalho B., Serrano K., Pereira A.D.S., Devine D.V., Acker J.P. (2015). Blood Bag Plasticizers Influence Red Blood Cell Vesiculation Rate without Altering the Lipid Composition of the Vesicles. Transfus. Med. Hemother..

[13] Lozano M., Cid J. (2013). DEHP plasticizer and blood bags: Challenges ahead. ISBT Sci. Ser..

[14] D’Alessandro A., Nemkov T., Hansen K.C. (2015). Rapid detection of DEHP in packed red blood cells stored under European and US standard conditions. Blood Transfus..

[15] Zarean M., Keikha M., Poursafa P., Khalighinejad P., Amin M., Kelishadi R. (2016). A systematic review on the adverse health effects of di-2-ethylhexyl phthalate. Environ. Sci. Pollut. Res..

[16] Tsai Y.-A., Tsai M.-S., Hou J.-W., Lin C.-L., Chen C.-Y., Chang C.-H., Liao K.-W., Wang S.-L., Chen B.-H., Wu M.-T. (2018). Evidence of high di(2-ethylhexyl) phthalate (DEHP) exposure due to tainted food intake in Taiwanese pregnant women and the health effects on birth outcomes. Sci. Total Environ..

[17] Tickner J.A., Schettler T., Guidotti T., McCally M., Rossi M. (2001). Health Risks Posed by Use of of Di-2-Ethylhexyl Phthalate (DEHP) in PVC Medical Devices: A Critical Review.pdf. Am. J. Ind. Med..

[18] Ferri M., Chiellini F. (2010). Materials Degradation in PVC Medical Devices, DEHP Leaching and Neonatal Outcomes. Curr. Med. Chem..

[19] Mersiowsky I. (2002). Long-term fate PVC in landfills. Prog. Polym. Sci..

[20] Zhang M., Buekens A., Jiang X., Li X. (2015). Dioxins and polyvinylchloride in combustion and fires. Waste Manag. Res..

[21] Van Vliet E.D.S., Reitano E.M., Chhabra J.S., Bergen G.P., Whyatt R.M. (2011). A review of alternatives to di (2-ethylhexyl) phthalate-containing medical devices in the neonatal intensive care unit. J. Perinatol..

[22] Malarvannan G., Onghena M., Verstraete S., van Puffelen E., Jacobs A., Vanhorebeek I., Verbruggen S.C., Joosten K.F., Berghe G.V.D., Jorens P.G. (2018). Phthalate and alternative plasticizers in indwelling medical devices in pediatric intensive care units. J. Hazard. Mater..

[23] Xie M., Wu Y., Little J.C., Marr L.C. (2015). Phthalates and alternative plasticizers and potential for contact exposure from children’s backpacks and toys. J. Expo. Sci. Environ. Epidemiol..

[24] (2010). Jegrelius, “Blood Bags—A pilot case to stimulate eco-innovation within the healthcare sector”, Jegrelius Institute for Applied Green Chemistry, Östersund, Sweden, Final Report—Vinnova Project Reg. No. 2008-0381, Oct. https://pvcfreebloodbag.eu/wp-content/uploads/2013/11/BloodBags_final_report_krcopy.pdf.

[25] Gulliksson H., Meinke S., Ravizza A., Larsson L., Höglund P. (2016). Storage of red blood cells in a novel polyolefin blood container: A pilot in vitro study. Vox Sang..

[26] Lagarón J.M., López-Rubio A., Fabra M.J. (2015). Poly(L-lactide)/ZnO nanocomposites as efficient UV-shielding coatings for packaging applications. J. Appl. Polym. Sci..

[27] Vesna O.B., Emi G.B., Veljko F. (2014). Compatibilization of thermoplastic polyurethane and polypropylene with a SEBS compatibilizer. Adv. Mater. Res..

[28] Banerjee S.S., Burbine S., Shivaprakash N.K., Mead J. (2019). 3D-printable PP/SEBS thermoplastic elastomeric blends: Preparation and properties. Polymers.

[29] Monti M., Scrivani M.T., Gianotti V. (2020). Effect of SEBS and OBC on the Impact Strength of Recycled Polypropylene/Talc Composites. Recycling.

[30] Lou C.-W., Huang C.-L., Pan Y.-J., Lin Z.-I., Song X.-M., Lin J.-H. (2016). Crystallization, mechanical, and electromagnetic properties of conductive polypropylene/SEBS composites. J. Polym. Res..

[31] Tjong S.C., Xu S.A., Li R.K.Y., Mai Y.W. (2002). Mechanical behavior and fracture toughness evaluation of maleic anhydride compatibilized short glass fiber / SEBS / polypropylene hybrid composites. Compos. Sci. Technol..

[32] Denac M., Musil V., Šmit I. (2005). Polypropylene/talc/SEBS (SEBS-g-MA) composites. Part 2. Mechanical properties. Compos. Part A Appl. Sci. Manuf..

[33] de Oliveira C.I.R., Rocha M.C.G., de Assis J.T., da Silva A.L.N. (2019). Morphological, mechanical, and thermal properties of PP/SEBS/talc composites. J. Thermoplast. Compos. Mater..

[34] Luo X., Chu H., Liu M. (2020). Synthesis of Bio-Plasticizer from Soybean Oil and Its Application in Poly (Vinyl Chloride) Films. J. Renew. Mater..

[35] Kormin S., Kormin F., Beg M.D.H. (2019). Effect of plasticizer on physical and mechanical properties of ldpe / sago starch blend Effect of plasticizer on physical and mechanical properties of ldpe / sago starch blend. J. Phys. Conf. Ser..

[36] Carmen R. (1993). The Selection of Plastic Materials for Blood Bags. Transfus. Med. Rev..

[37] Zahra N.M., Siswanto, Widiyanti P. (2018). The role of chitosan on polyvinyl chloride (PVC)-glycerol biocomposites for blood bag application. J. Biomim. Biomater. Biomed. Eng..

[38] Pötschke P., Pionteck J., Stutz H. (2002). Surface tension, interfacial tension, and morphology in blends of thermoplastic polyurethanes and polyolefins. Part I. Surface tension of melts of TPU model substances and polyolefins. Polymer.

[39] Balkan O., Demirer H., Kayali E.S. (2011). Effects of deformation rates on mechanical properties of PP/SEBS blends. J. Acheivement Mater. Manuf. Eng..

[40] Luna C.B.B., Siqueira D.D., Araújo E.M., do Nascimento E.P., da Costa Agra de Melo J.B. (2022). Evaluation of the SEBS copolymer in the compatibility of PP/ABS blends through mechanical, thermal, thermomechanical properties, and morphology. Polym. Adv. Technol..

[41] Luna C.B.B., Ferreira E.D.S.B., Siqueira D.D., da Silva W.A., Araújo E.M., Wellen R.M.R. (2019). Tailoring performance of PP/HIPS/SEBS through blending design. Mater. Res. Express.

[42] Chungprempree J., Charoenpongpool S., Preechawong J., Atthi N., Nithitanakul M. (2021). Simple preparation of polydimethylsiloxane and polyurethane blend film for marine antibiofouling application. Polymers.

[43] Liu C., Jiang S., Zhang S., Xi T., Sun Q., Xiong L. (2016). Characterization of edible corn starch nanocomposite films: The effect of self-assembled starch nanoparticles. Starch-Stärke.

[44] Kordjazi Z., Ajji A. (2020). Partially miscible polymer blends of ethyl cellulose and hydroxyl terminated polybutadiene. Polymer.

[45] Bourara H., Hadjout S., Benabdelghani Z., Etxeberria A. (2014). Miscibility and hydrogen bonding in blends of poly(4-vinylphenol)/Poly(vinyl methyl ketone). Polymers.

[46] Casadellà A., Schaetzle O., Loos K. (2016). Ammonium across a Selective Polymer Inclusion Membrane: Characterization, Transport, and Selectivity. Macromol. Rapid Commun..

[47] Chiu F.-C., Chu P.-H. (2006). Characterization of Solution-Mixed Polypropylene/Clay Nanocomposites without Compatibilizers. J. Polym. Res..

[48] Bao S., Tjong S.C. (2007). Impact essential work of fracture of polypropylene / montmorillonite nanocomposites toughened with SEBS-g-MA elastomer. Compos. Part A Appl. Sci. Manuf..

[49] Niu P., Liu B., Wei X., Wang X., Yang J. (2011). Study on mechanical properties and thermal stability of polypropylene/hemp fiber composites. J. Reinf. Plast. Compos..

[50] Taha T.A., Saleh A. (2018). Dynamic mechanical and optical characterization of PVC/fGO polymer nanocomposites. Appl. Phys. A.

[51] Bhavsar V., Tripathi D. (2018). Low and high frequency shielding effectiveness of PVC-PPy films. Polym. Bull..

[52] Abdelghany A., Meikhail M., Asker N. (2019). Synthesis and structural-biological correlation of PVC\PVAc polymer blends. J. Mater. Res. Technol..

[53] Matos M., Cordeiro R.A., Faneca H., Coelho J.F.J., Silvestre A.J.D., Sousa A.F. (2019). Replacing Di(2-ethylhexyl) terephthalate by Di(2-ethylhexyl) 2,5-furandicarboxylate for PVC plasticization: Synthesis, materials preparation and characterization. Materials.

[54] Gohatre O.K., Biswal M., Mohanty S., Nayak S.K. (2020). Study on thermal, mechanical and morphological properties of recycled poly(vinyl chloride)/fly ash composites. Polym. Int..

[55] Miyamoto M., Sasakawa S. (1988). Effects of Autoclave Sterilization on the Physical Properties of Storage Bags and Granulocyte Function. Vox Sang..

[56] Eades B. (2020). Freezing and recovering rare red blood cells using glycerol. Immunohematology.

[57] Park M.-H. (2015). Recovery of the mechanical properties of recycled styrene-ethylene-butylene-styrene/polypropylene (SEBS/PP) composites. Toxicol. Environ. Health Sci..

[58] Corona-Galván S., Henche A.G., Alvarez L.O., Dynasol G. (2020). Developments in SEBS for Medical and Other Valuable Applications. Rubber World Tech. Mag..

[59] Kim J.K., Kim C.H., Park M.H. (2016). Effects of multiple recycling on the structure and morphology of SEBS/PP composites. Bull. Korean Chem. Soc..

[60] Sampson J., De Korte D. (2011). DEHP-plasticised PVC: Relevance to blood services. Transfus. Med..

[61] Mazitova A.K., Vikhareva I.N., Aminova G.K., Savicheva J.N. (2020). Application of Zinc Oxide to Obtain and Modify Properties of Adipate Plasticizer of Polyvinyl Chloride. Polymers.

[62] Bhunia K., Sablani S.S., Tang J., Rasco B. (2013). Migration of Chemical Compounds from Packaging Polymers during Microwave, Conventional Heat Treatment, and Storage. Compr. Rev. Food Sci. Food Saf..

[63] European Commission “Commission Regulation (EU) No. 10/2011 on Plastic Materials and Articles Intended to Come into Contact with Food (14 January 2011)”, Official Journal of the European Union. https://eur-lex.europa.eu/LexUriServ/LexUriServ.do?uri=OJ:L:2011:012:0001:0089:en:PD.

[64] Bodecchi L.M., Durante C., Malagoli M., Manfredini M., Marchetti A., Sighinolfi S. (2011). Distribution of Heat Stabilizers in Plasticized PVC-Based Biomedical Devices: Temperature and Time Effects. Int. J. Spectrosc..

