# Supplementary material for: Study on the Physical, Thermal and Mechanical Properties of SEBS/PP (Styrene-Ethylene-Butylene-Styrene/Polypropylene) Blend as a Medical Fluid Bag
Source: Polymers (Basel). 2022 Aug 11;14(16):3267. doi: 10.3390/polym14163267 (PMC9416621; doi:10.3390/polym14163267)
Supplement: Supplementary file 1 [file polymers-14-03267-s001.zip › polymers-1812702-supplementary.pdf]

Supplementary data

Table S1: Mass of the SEBS/PP 50/50 specimens before and after immersion in distilled water

SEBS/PP 50/50	Mass of specimen before immersion (g)	Mass of specimen after immersion (g)	Water absorption (%)
Specimen 1	0.26	0.26	0
Specimen 2	0.3	0.3	0
Specimen 3	0.3	0.3	0
Specimen 4	0.28	0.28	0
Specimen 5	0.25	0.25	0

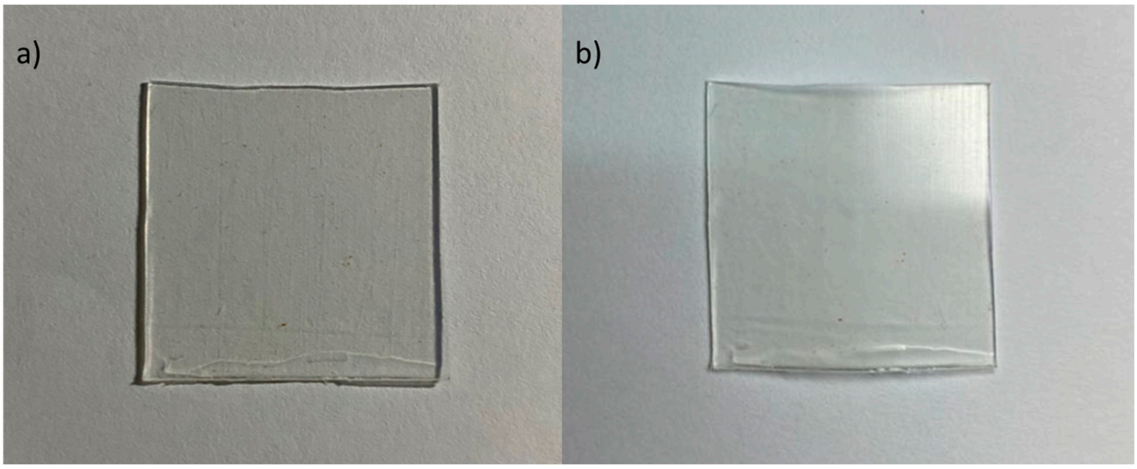

Figure S1: Figure of the SEBS/PP 50/50 specimen (a) before steam sterilisation and (b) after steam sterilisation.
